# Mental health of indigenous school children in Northern Chile

**DOI:** 10.1186/1471-244X-14-11

**Published:** 2014-01-17

**Authors:** Alejandra Caqueo-Urízar, Alfonso Urzúa, Koen De Munter

**Affiliations:** 1 Departamento de Filosofía y Psicología, Universidad de Tarapacá, Avenida 18 de Septiembre #2222, Arica, Chile; 2 Escuela de Psicología, Universidad Católica del Norte, Avenida Angamos, 0610 Antofagasta, Chile; 3 Departamento de Antropología, Universidad Alberto Hurtado, Cienfuegos 41, Santiago, Chile

**Keywords:** Mental health, Indigenous, Aymara children, Anxiety, Depression

## Abstract

**Background:**

Anxiety and depressive disorders occur in all stages of life and are the most common childhood disorders. However, only recently has attention been paid to mental health problems in indigenous children and studies of anxiety and depressive disorders in these children are still scarce. This study compares the prevalence of anxiety and depressive symptoms in Aymara and non-Aymara children. Among the Aymara children, the study examines the relations between these symptoms and the degree of involvement with Aymara culture.

**Methods:**

We recruited 748 children aged 9 to 15 years from nine schools serving low socioeconomic classes in the city of Arica, in northern Chile. The children were equally divided between boys and girls and 37% of the children were Aymara. To evaluate anxiety and depressive symptoms we used the Stress in Children (SiC) instrument and the Children Depression Inventory-Short version (CDI-S), and used an instrument we developed to assess level of involvement in the Aymara culture.

**Results:**

There was no significant difference between Aymara and non-Aymara children on any of the instrument scales. Dividing the Aymara children into high-involvement (*n* = 89) and low-involvement (*n* = 186) groups, the low-involvement group had significantly higher scores on the *Hopelessness* subscale of the CDI-S (*p* = 0.02) and scores of marginally higher significance in overall *Anxiety* on the SiC (*p =* 0.06).

**Conclusions:**

Although Aymara children have migrated from the high Andean plateau to the city, this migration has not resulted in a greater presence of anxiety and depressive symptoms. Greater involvement with the Aymara culture may be a protective factor against anxiety and depressive symptoms in Aymara children. This point to an additional benefit of maintaining cultural traditions within this population.

## Background

Psychiatric disorders have a high prevalence in children and adolescents, and often persistent into adulthood. Their importance has made them a growing subject of research in recent years [1]. Among the psychiatric disorders, anxiety and depression are the most common reasons for seeking mental health services [2-5]. Worlwide statistics reported rates of anxiety in children and adolescents range from 5.6% to 21%, depending on the criteria and the type of anxiety disorder [2,6-9], with higher rates observed in older children [10]. The prevalence of depressive disorders also varies depending on the population and evaluation method [11]. Rates of depressive disorders between 0.5% and 2.0% have been reported for children in the general population aged 9 to 11 years [8].

In Chile, anxiety and depressive disorders are the most prevalent mental health diagnoses in children, with reported rates of 8.3% and 5.1%, respectively [12].

There has been very little research on anxiety and depression among different ethnic groups, with no research in Chile, despite its numerous indigenous groups.

Stressors that can impact mental health, such as racism, family disconnectedness, community dysfunction, and social disadvantage, are more prevalent among ethnic minorities [13,14]. Indigenous peoples have identified certain stressors as causes of poor health, such as loss of native lands, culture, and identity; covert and overt racism; marginalization; and powerlessness [14-17].

Culture can also affect the way in which symptoms of mental illness manifest. Culture can determine or frame causative, precipitating, or maintenance factors that influence the onset, symptom profile, impact, course, and outcome of mental illness [17,18].

The prevalence of anxiety symptoms in children and adolescents also varies between ethnic minorities [19]. For instance, adolescent immigrants in Belgium reported more traumatic experiences, more problems with their peers, and greater avoidance than non-immigrants [20]. Similar findings were observed in a study of immigrant children in the Netherlands, who demonstrated a greater level of externalized and internalized problems than their peers [21-24].

Migration to a new country or sociocultural context can cause stress due to cultural acclimatization. Such stress tends to increase levels of anxiety and depression, loneliness, psychosomatic symptoms, and contribute to a confused sense of identity [25].

Research on stress due to cultural acclimatization has focused on how conflicts with the host language and the perception of discrimination can affect psychological well-being [26,27]. However, stress due to cultural acclimatization can take various paths. Thus, acclimatization is driven by transcultural or intercultural dynamics [28,29].

While there are numerous studies on the mental health of indigenous adults and adolescents in North America, studies of indigenous children are rare, and even more rare among indigenous children of Latin America [30-34].

The lack of such studies on Chilean indigenous children is worrisome. Mental health problems in childhood have important repercussions in adulthood, including declines in academic achievement and interpersonal relations, as well as ongoing behavioural problems and drug abuse [11,35,36]. Factors pertaining to the risks to and protection of mental health in children and adolescents vary in different contexts, especially between developed and developing countries [37].

This study’s objective is to analyze the differences in the presence of anxiety and depressive symptoms between Aymara and non-Aymara children and to evaluate the relation between mental disorders and cultural involvement.

Aymara is a centuries-old culture centred in the Andes mountains. In 2012, the Aymara community had a population of about 2 million living in g central and western Bolivia, southern Peru, northern Chile, and north western Argentina [38,39]. The Aymara have an agricultural economy based on cultivation of potatoes, corn, and quinoa and domestication of llamas, alpacas, and vicuñas [38,40,41], two activities that are complementary, both ecologically and economically [42,43]. The Aymara is a geographically broad and heterogeneous group, although certain common characteristics undoubtedly prevail [44]. The culture is characterized by its intergenerational communication, where elders provide advice to the young. In addition, it is a culture in which the mother focuses on household tasks and education, the father makes the family and monetary decisions and is the breadwinner, and family members work together to complete various tasks, with young children helping out with simple household tasks.

The Aymara community in Chile has a population of approximately 48,000 [45], only 2,300 of which still live in their original mountain territories. The rest have emigrated towards the nearby port cities and mining regions, where they have intermingled with the working classes from other areas of the country [38,46-48]. Chile’s large-scale migration towards the coast, its policy of Chilenization – where indigenous people are encouraged to accept Chilean culture and abandon their own particularly during the Pinochet dictatorship – this last forged an identity for the Aymara people, shaped by the difficulty and complexity of the process in different areas [49].

This mass migration and rapid abandonment of rural settlements in the Andean foothills have been among the most difficult experiences for the Aymara. Migration has been a complex phenomenon that has not necessarily involved a departure without return, as evidenced by the number of simultaneous residencies and linkages that are maintained with the native communities [50].

In adapting to a hegemonic culture, Aymara families have abandoned, to some extent, traditional cultural patterns and are slowly adopting new and increasingly intercultural lifestyles [51,52]. These intercultural dynamics have led to an identity crisis, not only at the level of the Aymara population, but at the level of the country, in which the issue of interculturalism has not yet been constructively addressed. Given this context, a large number of people who could be identified as Aymara by heritage or because they still practice certain Aymara customs, are no longer considered such, at least until recently [53].

Various government organizations around the world have expressed concern for the rights of indigenous people, especially their autonomy to raise, educate, and ensure the well-being of their children, in line with children’s rights [54]. Such recognition, however, is insufficient to eliminate the discrimination and other problems Aymara descendants face, leading to reduced access to economic, educational, and social opportunities.

Given these inequalities and the stress due to cultural acclimatization experienced by Aymara youth, we expect higher levels of anxiety and depressive symptoms among Aymara children compared to non-Aymara children.

## Methods

### Participants

The sample consisted of 748 Chilean children aged 9 to 15 years (mean 11.81, SD 1.41). They were recruited from the fifth to eighth grades of nine elementary schools serving lower socioeconomic classes in the city of Arica, which has a large Aymara population. Four of the schools were public and five were government-subsidized private schools. The children were equally divided between boys (*n* = 374) and girls (*n* = 374) and 275 (37%) of the children were Aymara. Of the total sample there were 64 cases missing, this because they did not complete questionnaires. The city and schools were chosen in an effort to maximize the number of Aymara included in the study.

### Instruments

***Stress in Children (SiC)*** [55]: SiC is a self-administered instrument for children aged 9 to 12 years. It is designed to measure perceived anxiety and low levels of well-being, in addition to important aspects relating to confrontation and social support. These factors may be considered part the broader concept of subjective health. This instrument was adapted for Chile by Caqueo-Urízar, Urzúa, and Osika [56].

The questionnaire consists of 21 items scored on a four-point Likert scale, with zero standing for none, one for sometimes, two for almost always, and three for always. The overall score is obtained by summing the points from each item, with a higher score indicating a higher degree of perceived stress. The mean plus or minus the standard deviation in a normal Swedish population was 2.05 ± 0.41 (girls, 1.99 ± 0.42; boys, 2.15 ± 0.37), with no significant differences detected between the genders. The two subscales of the questionnaire measure 1) loss of well-being, with items such as ‘I feel lonely’ and ‘I get sad’, and 2) sources of distress, requiring responses dealing with daily stressors, school being one of those with the highest factor loading. This questionnaire has high internal consistency (Cronbach’s alpha = 0.86) and is strongly associated with the Beck Youth Inventories of Emotional and Social Impairment [57].

***Children Depression Inventory-Short (CDI-S)*** [58]: The CDI-S measures depressive symptoms in children. It contains three subscales: self-esteem, anhedonia, and hopelessness. It was adapted into Spanish by Barrio et al. [59]. The instrument contains 10 items that are easy for children to complete. It uses a three-point scale indicating absent, mild, or definite symptoms. Its reliability is good, with Cronbach’s alpha ranging from 0.71 to 0.89 and test–retest coefficients ranging from 0.74 to 0.83. This measure’s construct validity has also been confirmed [60]. Our sample’s Cronbach’s alpha was 0.84. The CDI-S can be used individually or collectively for subjects aged 7 to 17 years and takes between five and seven minutes to administer. The scores ranged from zero to 20, with an overall mean of 2.82 and a standard deviation of 2.43. The cut-off point is 7.04 for subjects aged 9 to 11 years and 8.24 for subjects aged 12 to 15 years.

***Inventory of the Level of Involvement in Aymara Culture – Escala de Involucramiento en la Cultura Aymara (EICA)*** [61]: This instrument measures a child’s level of involvement in Aymara culture. The development of this scale was carried out by the authors of the study who an epidemiologist, a clinical psychologist and an anthropologist. The instrument was not based on another scale, since there is no information and was held in two stages, one qualitative and one quantitative. It contains 25 items evaluated on a Likert scale from zero to two, where the higher the score, the greater the role of Aymara culture in the daily life of the youth. These scores correspond to the average number of points obtained in the scale or respective subscale after multiplication by 10 to avoid many decimal places and they represent the degree of involvement in each of several aspects of Aymara culture, ranging from zero to 20 points. The scale is composed of six subscales: family language use (six items), personal use of the Aymara language (three items), traditional celebrations (five items), traditional employment (three items), dances (five items), and music (three items). The scale has adequate psychometric properties and helps identify both children who are and children who are not highly involved in Aymara culture. The scale has a Cronbach’s alpha of 0.91.

### Procedure

The study was approved by the Ethics Committee of the University of Tarapacá and the National Commission on Science and Technology of the Government of Chile (CONICYT). The project complies with the Declaration of Helsinki [62].

The study used an inventory of public and state-subsidized private schools. The latter have a considerable population of students of Aymara origin, according to the list of beneficiaries of the Indigenous Scholarship for 2010–2011, granted by the National Council for School Assistance and Grants for the Ministry of Education. The schools were chosen and their principals contacted to obtain their consent to participate in the study. Once approval was received, the children were invited to participate in the study. Parents were asked to sign an informed consent form consenting for their children to participate in the study. Children with consenting parents were free to decide for themselves whether to participate in the study.

The children were assessed during school hours in group sessions run by two psychologists. The criteria for including a child in the Aymara arm of the study were:

– One or more Aymara surnames, as established by the Indigenous Law;

– Self-description as Aymara ethnicity, implying both the child and the parents consider themselves indigenous.

### Data analysis

Statistical analysis of survey data was performed using SPSS 17.0. Means and standard deviations were calculated for subscale scores of each group and an independent samples t-test was run.

## Results

First, our analysis considered the information on ethnicity provided by the parents. As shown in Table 1, there was no significant difference in anxiety or depression symptoms between the Aymara and non-Aymara groups.

**Table 1:** Anxiety and depression symptoms in Aymara and non-Aymara children, measured by the SiC and CDI-S

**Instrument**	**Subscale**	**Aymara (*n*** **= 275)**		**Non-Aymara (*n*** **= 409)**		***t*-Test**	***p*-Value**
		**Mean**	**SD**	**Mean**	**SD**		
SiC	Loss of well-being	5.44	3.59	5.06	3.17	1.38	0.16
	Source of stress	7.78	3.73	7.69	3.79	0.29	0.77
	Total anxiety	13.24	6.04	12.76	5.97	1.01	0.30
CDI-S	Self-esteem	1.57	1.57	1.49	1.49	0.70	0.48
	Anhedonia	1.12	1.36	1.08	1.21	0.42	0.67
	Hopelessness	1.38	1.24	1.27	1.09	1.23	0.21
	Total depression	4.08	3.26	3.85	2.99	0.94	0.34

*N* = sample; X = Mean; SD = Standard Deviation.

The EICA, which was administered only to the Aymara children, categorized 89 children as having high involvement with Aymara culture and 186 children as having low involvement. Table 2 shows the SiC and CDI-S results for these two subgroups. There was a significant difference between the subgroups on the hopelessness subscale of the CDI-S (*p* = 0.02) and a marginally significant difference on the overall score on the SiC (*p* = 0.06). In both these cases, the low-involvement group scored higher.

**Table 2:** Anxiety and depression symptoms in Aymara children with high and low levels of cultural involvement

**Instrument**	**Subscale**	**High involvement (*n*** **= 89)**		**Low involvement (*n*** **= 186)**		***t*-Test**	***p*-Value**
		**Mean**	**SD**	**Mean**	**SD**		
SiC	Loss of well-being	5.04	3.39	5.62	3.68	1.26	0.20
	Source of stress	7.23	3.54	8.04	3.81	1.69	0.09
	Total anxiety	12.28	5.39	13.70	6.28	1.80	0.06
CDI-S	Self-esteem	1.49	1.43	1.61	1.64	0.61	0.54
	Anhedonia	1.16	1.24	1.10	1.42	−0.33	0.73
	Hopelessness	1.15	1.08	1.50	1.29	2.15	0.02
	Total depression	3.82	2.81	4.21	3.45	0.94	0.34

*N* = sample; X = Mean; SD = Standard Deviation.

## Discussion

The study’s objective was to measure potential differences in the mental health of Aymara and non-Aymara children in northern Chile. We found no difference in levels of anxiety or depression symptoms between Aymara children and their non-Aymara peers. This finding is contrary to the stress due to cultural acclimatization hypothesis [25]. This suggests that Aymara children possess adequate adaptive mechanisms for integrating into an urban context. These results are consistent with those of Zwirs et al. [63] in Europe and Costello et al. [64] in the United States.

While there may be no difference in anxiety and depression symptoms between Aymara children and their non-Aymara peers, differences could still arise as they grow into adulthood and encounter greater discrimination [63].

Another possibility is that although these particular children are Aymara, they may have been away from the high Andean plateau long enough to have learned to live between two cultures: that of their ancestors and their current urban environment. It is possible that stress from cultural acclimatization may not have developed in these children and consequently there is no reduction in their mental health. In fact, various aspects of their lives may have improved [65]. Some authors describe the ‘cultural advantages’ of younger groups ‘forced’ into historic processes of cultural acclimatization [66]. This notion is consistent with a study of immigrant children in Barcelona, who developed strategies for dealing with the adaptation process and actively searched for solutions to conflicts [67].

In anthropological terms, the dynamics of cultural acclimatization can result on difficult but constructive processes of ‘creolization’. This means that ‘contact zones’ – interfaces between different practices or cultural environments [68] – are being generated in these indigenous children [66]. Outside these zones, coping strategies can arise that allow individuals to combine different living strategies, enabling adequate mental health.

Within the Aymara children, those with high involvement in Aymara traditions exhibited lower levels of anxiety and fewer feelings of hopelessness. These results indicate that Aymara children with high cultural involvement may cope better with anxiety and feelings of hopelessness. Traditional celebrations, for example, have two characteristics that protect against the development of mental disorders: social and community participation and religious events. The high involvement children’s regular contact with other children of the community involves them more with the cultural perception of more positive events that happen in one’s life, with fewer feelings of hopelessness. Research during the past two decades has shown that social support is significantly linked to psychological well-being [69]. Studies demonstrate that children with social support who are exposed to adverse experiences exhibit less psychological illness compared to children without social support [69].

Religious beliefs have also been associated with healthy adjustment in adolescence. This may function through personal beliefs regarding behaviour, constraints on behaviour, or support for healthy behaviour by religious institutions [70,71]. In our study, participation in activities linked to religious beliefs or ritual practices specific to the Aymara culture could be a protective factor.

The study has a number of limitations. First, selection of the subjects was not random. Second, only Aymara children in Chile were evaluated, and these results may not apply to Aymara in other countries. Future studies should also examine children of other ethnicities in Chile, such as Mapuches, Rapa Nuis, and Quechuas, as well as indigenous children in other countries. Third, neither the SiC nor the CDI-S were validated in Chile, and only the SiC was adapted to the country. There is a likelihood of difficulties arising when psychological concepts and measurement techniques developed for one culture are used in another [20,72]. This issue can also be related to social expectancy. Fourth, this study contains no information from parents or teachers, which would have been important to consider to properly understanding the children’s problems. Finally, this was a cross-sectional study. It is important to carry out longitudinal studies to evaluate the consistency of findings over time [73].

## Conclusions

In our study population, Aymara school children living in the city did not differ significantly from their non-Aymara peers in levels of anxiety or depressive symptoms. Among Aymara children, greater involvement with their culture conferred some protection against anxiety and depressive symptoms. This points to an additional benefit of maintaining cultural traditions within this population.

## Abbreviations

SiC: Stress in Children; CDI-S: Children Depression Inventory-Short; EICA: Inventory of the Level of Involvement in the Aymara Culture (Escala de Involucramiento en la Cultura Aymara).

## Competing interests

The authors have declared that there are no conflicts of interest in relation to the subject of this study.

## Authors’ contributions

ACU contributed to the design and coordination of the study. AU was responsible for the primary study design, consulted on the methodology, and assisted with the data analysis and interpretation. KDM participated in the data collection and manuscript editing. All authors read and approved the final manuscript.

## Pre-publication history

The pre-publication history for this paper can be accessed here:

http://www.biomedcentral.com/1471-244X/14/11/prepub
